# Design of a flow modulation device to facilitate individualized ventilation in a shared ventilator setup

**DOI:** 10.1007/s10877-024-01138-1

**Published:** 2024-04-01

**Authors:** Michiel Stiers, Jan Vercauteren, Tom Schepens, Matthias Mergeay, Luc Janssen, Olivier Hoogmartens, Arne Neyrinck, Benoît G. Marinus, Marc Sabbe

**Affiliations:** 1 Department of Public Health and Primary Care, Research unit Emergency Medicine, KU Leuven, 3000 Leuven, Belgium; 2 Department of Emergency Medicine, University Hospitals Leuven, Herestraat 49, 3000 Leuven, Belgium; 3 Department of Mechanical Engineering, Royal Military Academy, Renaissancelaan 30, Brussels, Belgium; 4 Department of Intensive Care Medicine, Ghent University Hospital, C Heymanslaan 10, Ghent, Belgium; 5 Department of Anesthesiology and Critical Care Medicine, St-Dimpna, J.-B. Stessensstraat 2, 2440 Geel, Belgium; 6 Department of Cardiovascular Sciences, Research unit Anesthesiology and Algology, KU Leuven, 3000 Leuven, Belgium; 7 Department of Anesthesiology, University Hospitals Leuven, Herestraat 49, 3000 Leuven, Belgium

**Keywords:** Ventilator, Surge capacity, Flow modulator, Medical device, Individualized shared ventilation

## Abstract

This study aims to resolve the unmet need for ventilator surge capacity by developing a prototype device that can alter patient-specific flow in a shared ventilator setup. The device is designed to deliver a predictable tidal volume (VT), requiring minimal additional monitoring and workload. The prototyped device was tested in an in vitro bench setup for its performance against the intended use and design criteria. The ventilation parameters: VT and airway pressures, and ventilation profiles: pressure, flow and volume were measured for different ventilator and device settings for a healthy and ARDS simulated lung pathology. We obtained VTs with a linear correlation with valve openings from 10 to 100% across set inspiratory pressures (IPs) of 20 to 30 cmH_2_O. Airway pressure varied with valve opening and lung elastance but did not exceed set IPs. Performance was consistent in both healthy and ARDS-simulated lung conditions. The ventilation profile diverged from traditional pressure-controlled profiles. We present the design a flow modulator to titrate VTs in a shared ventilator setup. Application of the flow modulator resulted in a characteristic flow profile that differs from pressure- or volume controlled ventilation. The development of the flow modulator enables further validation of the Individualized Shared Ventilation (ISV) technology with individualization of delivered VTs and the development of a clinical protocol facilitating its clinical use during a ventilator surge capacity problem.

**Supplementary Information:**

The online version contains supplementary material available at 10.1007/s10877-024-01138-1.

## Background

The scenario of a potential shortage of ventilators, a ventilator surge capacity problem, changed from a mere theoretical consideration to a reality during the COVID-19 pandemic [1]. Solutions were proposed worldwide, ranging from ad hoc assembly of automatic resuscitators to patient allocation protocols [2, 3]. The concept of shared ventilation, where multiple patients share the same ventilator, has been widely discussed. It has triggered renewed research and was even applied during the surge in the United States [2, 4]. Several causes can give rise to mass casualty respiratory failure, ranging from bio-terrorism involving a nerve agent to natural disasters. Mechanical ventilation can be a bottleneck for mortality in acute respiratory failure. Several factors are crucial in this context, including the number of patients, the onset and development of Acute Respiratory Distress Syndrome (ARDS), and the duration of morbidity [1]. Although Neyman and Irvin are commonly cited first to have discussed shared ventilation [5], the original idea to increase the patient capacity of a ventilator was first described in 1994 by Sommer et al. with an anaesthesia-style, bag-in-the-box system in a limited proof-of-concept [6]. In 2002, a patent was granted to Lerner for ‘multiplex ventilation’, a system that could ventilate up to eight patients with a single gas source, again without further scientific elaboration or described applications [7]. In the original 2006 paper, Neyman and Irvin discussed a configuration in which four ventilation circuits with two splitters were connected to one ventilator to ventilate four artificial lungs [5]. This proof-of-concept was used in an in vivo pilot study by Paladino et al. in 2008 to ventilate four sheep for 12 h [8]. These preliminary results were framed within the limitations, risks and potential danger of oversimplification of this proof-of-concept and demonstrated in an initial bench study by Branson et al. [9–11] However, despite some evidence supporting the potential of a shared ventilator setup, a joint statement against the concept of shared ventilation was released in 2020. The arguments concerned ethical considerations, safety and feasibility aspects [12]. 

**Fig. 1:**
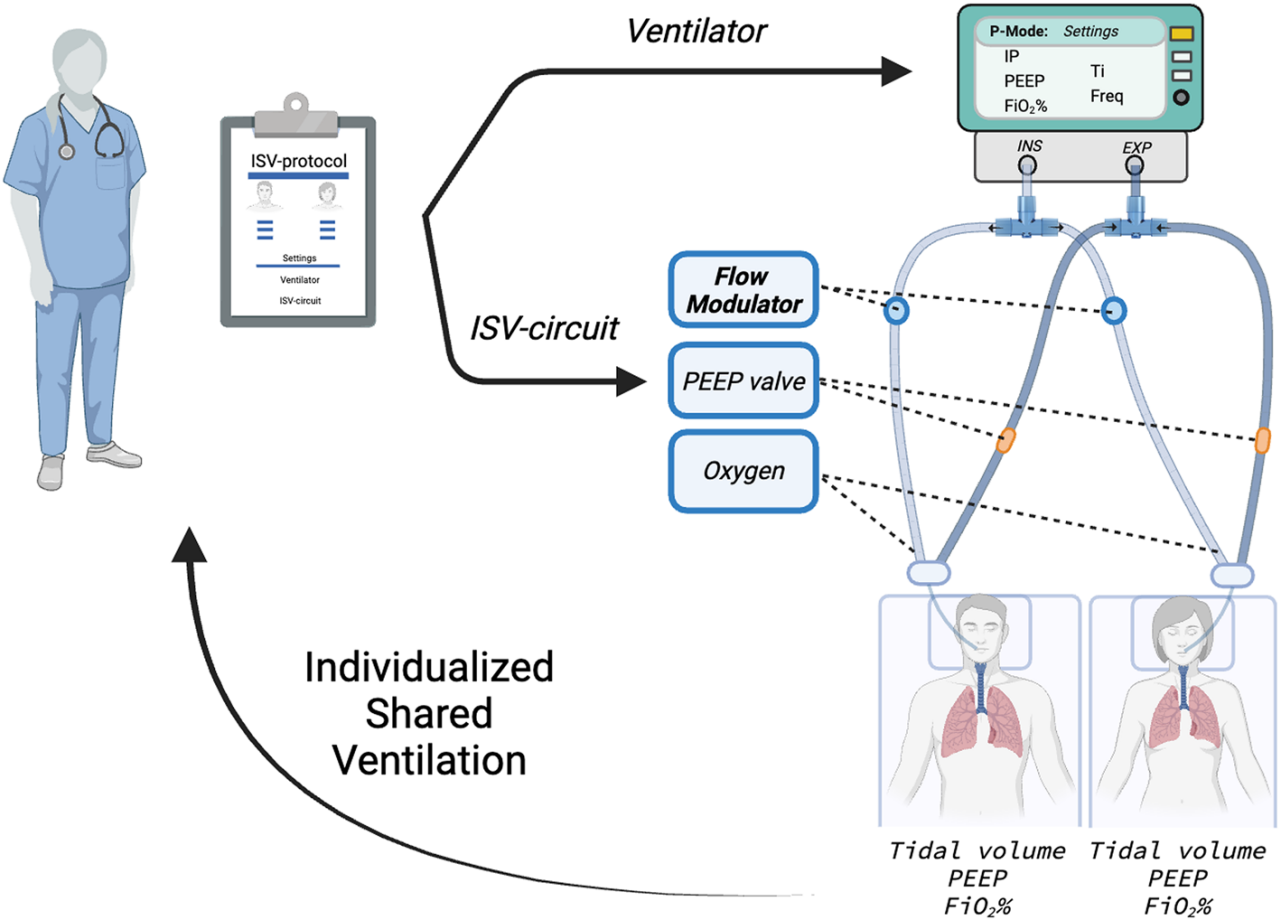
Schematic illustration of Individualized Shared Ventilation (ISV) setup. The ISV system operates with a ventilator in pressure-controlled mode. It features a split inspiratory (INS) and expiratory (EXP) circuit, facilitated by two T-pieces, four one-way valves, and two heat and moisture exchange filters, directing airflow to each patient. Within the inspiratory circuit for each patient, tidal volumes (VT) are individualized by a prototyped device, the flow modulator. On the expiratory circuit, an inline Positive End-Expiratory Pressure (PEEP) valve allows for patient-specific PEEP settings. Oxygen concentration can be individualized through an additional oxygen flow in the respective patient’s inspiratory circuit. The ISV system enables individualized ventilation parameters for each patient, including desired VT, PEEP, and Fraction of Inspired Oxygen (FiO_2_%), independent of the contralateral patient. Each ISV breath cycle exhibits a unique pressure, flow, and volume profile. Physicians can achieve the desired ventilation parameters by configuring the ventilator and ISV circuit according to a predefined ISV protocol. Created using BioRender.com

One of the setbacks of a shared ventilator setup, the naive system, is that patients need to be paired by similar ideal body weight and respiratory mechanics (lung compliance and airway resistance) and cannot be dynamically managed over time. This naïve system was then optimized during the innovative momentum of the pandemic [13, 14]. The innovative research tackling these shortcomings and improving safety and feasibility, has led to a new field of circuit modifications. The concept of Individualized Shared Ventilation (ISV), in which the ventilation of two different patients can be individualized and adapted through a modified ventilator circuit to enable lung protective ventilation was established [15–29]. The ISV circuit enables the titration of each patient’s Tidal Volume (VT), Positive End-Expiratory Pressure (PEEP), and Fraction of Inspired Oxygen (FiO_2_%). (Fig. 1) This allows to adapt ventilation to the patient, and not the other way around, as in the naive system. Additionally, two heat and moisture exchange filters (HMEFs) and four one-way valves should be provided to prevent cross contamination and pendelluft [30, 31]. The ventilator must be used in a pressure-controlled mode. As in volume-controlled mode, any change in impedance (compliance, resistance) of the patient or of the respiratory circuit will have a major effect on volume distribution [20, 22, 25, 30]. 

To individualize the VT, two types of valves were described and integrated into a variety of devices [25]. On the one hand, flow control valves (FCV) influence the cross-sectional area and consequently the tubing resistance. This was achieved by various valve types, including needle, ball and diaphragm valves. On the other hand, there are pressure-relief valves (PRV) that open when a certain pressure is exceeded and remain closed if not. This was achieved by a spring load. The performance of these devices is crucial for clinical implementation and the development of a clinical protocol [22]. The need for additional monitoring and caring staff workload increases if these devices deliver unpredictable or difficult-to-adjust VTs. Furthermore, the risk for ventilator induced lung injury (VILI) increases when these devices are inaccurate or poorly refined, or if a small adjustment on the device induces large changes in VT.

To decrease the barrier to clinical implementation and to maximize patient safety, ISV needs to deliver appropriate medical care. Therefore, there was a clear unmet medical need for a device capable of accurately and predictably individualizing VTs during ISV for different lung pathologies. The primary aim of this study was to develop a prototype valve in bench with predictable generated VTs and with small incremental steps within a clinically relevant range. This study covers the research and development phase of a new prototyped device, its performance testing and the description of its ventilation profiles during ISV in a bench setup.

## Methods

### ISV bench setup

**Fig. 2:**
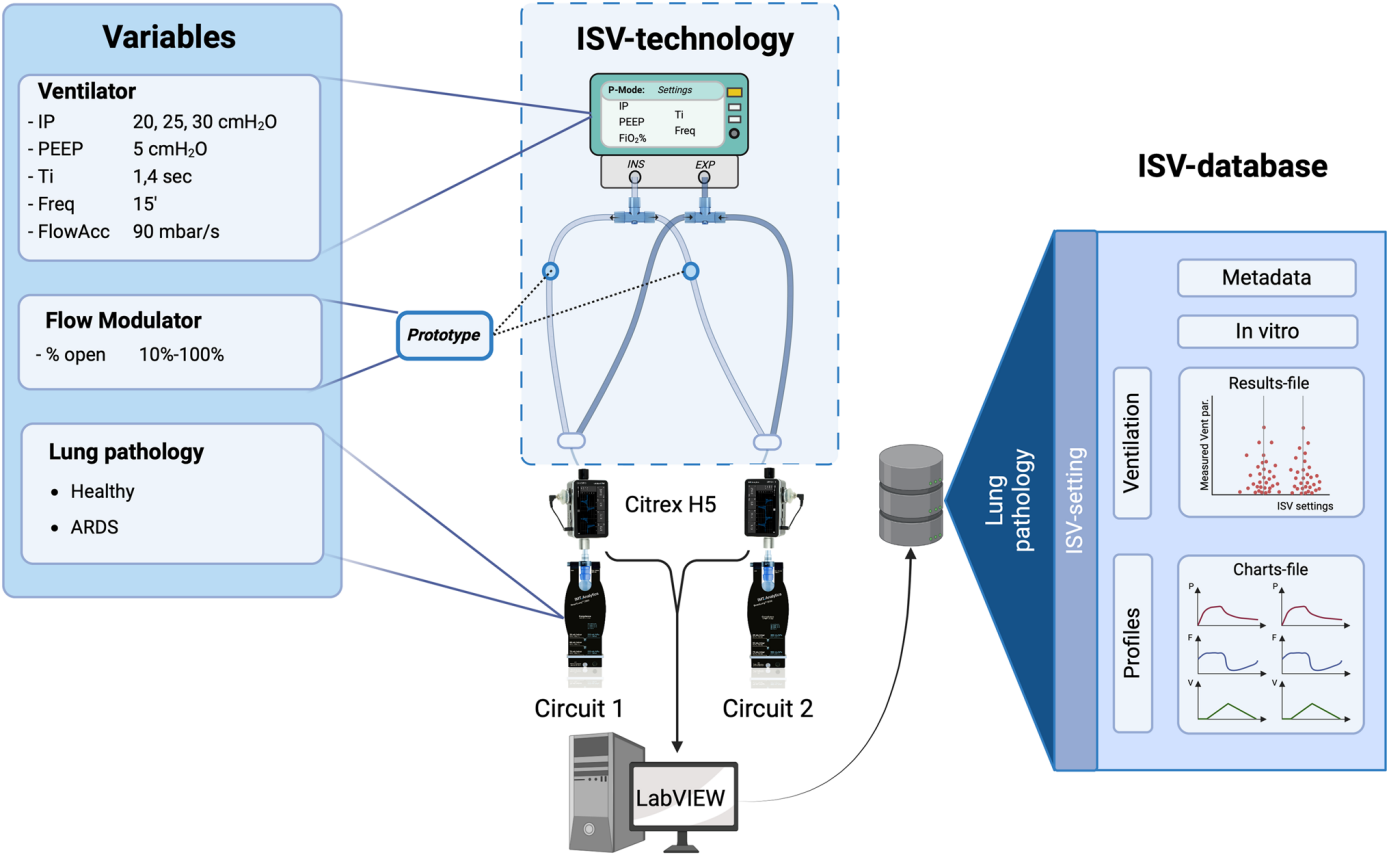
Schematic representation of the in vitro Individualized Shared Ventilation (ISV) bench setup. The configuration comprises an Intensive Care Unit (ICU) ventilator (Savina Dräger) operating in pressure-controlled mode, integrated with an ISV circuit. Within this circuit, a prototype flow modulator is inserted on the inspiratory limb. This assembly is connected to a Citrex H5 measuring device, which is subsequently interfaced with LabVIEW software. The configurations for both the ventilator and the flow modulator were established based on the protocols of the various bench tests. Test lungs are employed to simulate both healthy and Acute Respiratory Distress Syndrome (ARDS) lung pathologies in circuit 1, featuring an airway resistance of 5 mbar/L/s and respective compliances of 60 and 25 mL/mbar. In circuit 2 the test lung always had a fixed airway resistance of 5 mbar/L/s and  compliance of 60 mL/mbar. Data and associated metadata for each ISV ventilation cycle were collected, categorized by lung pathology and specific ISV settings. This includes ventilation parameters as functions of the ISV settings, as well as ventilation profiles for pressure, flow, and volume. Created using BioRender.com

The ISV bench setup, as described in Fig. 2, consisted of an ICU-ventilator Dräger Savina 300 (Dräger, Lübeck, Germany), set in a pressure-control mode (PC-AC) for different set inspiratory pressures. The ISV-circuit was attached and made from standard adult 22 mm tubes, two HMEFs, two T-pieces and four one-way valves (Intersurgical, Berkshire, UK). The lung pathology was simulated with two artificial test lungs, Smart Lung 2000 (IMT analytics, Buchs, Switzerland) by setting their airway resistance and elastance. The prototyped device was placed in each inspiratory part of the ISV-circuit while in front of each test lung an ISO certified measuring device, Citrex H5 (IMT analytics, Buchs, Switzerland) was inserted to measure the pressure drop across the prototyped device, the airway pressure and flow, and calculate the ventilation parameters. Data collection was facilitated by a National Instruments PXI system, utilizing LabVIEW software (National Instruments, Austin, TX, USA). The system interfaced with the Citrex H5 via a serial connection, sampling data at a frequency of 50 Hz. Following quality control by the researcher, the data, along with associated metadata, were stored in a dedicated database. Each data file is assigned a unique test-ID and encompasses distinct tidal cycles, all of which maintain consistent settings for the ventilator, prototyped device, and test lungs.

### Prototype design and development

The objective of the bench phase was to design and develop a prototype valve. This medical device was intended to meet specific design criteria and the intended use. The intended use of the device was defined as to individually regulate VTs in an ISV ventilator circuit, with an intensive care unit (ICU) ventilator in a pressure-controlled mode, when a ventilator surge capacity problem occurs. Once integrated into the ISV setup, the device will enable a physician to ventilate two patients simultaneously, independently, and accurately. Furthermore, it will allow for the management of their ventilation requirements over time using a single ventilator (Fig. 1).

Furthermore, we a priori defined that the device should meet the following design creteria: the device should allow a predictable and accurate regulation of VTs by manipulating airflow with a rotatory element. The ideal scenario would involve a linear relationship between the device’s settings and the resulting VT. This regulation should be achieved with small incremental steps within a clinically relevant VT range. The clinically relevant range for an adult was defined based on a 30 kg minimum weight and a lung protective ventilation strategy using VT ranging from 6 to 8 mL/kg ideal body weight, resulting in a clinically relevant VT range of 180 mL to 720 mL. Additionally, the device should be designed for ease of independent operation. Modulations on the device should only affect the ipsilateral patient. Finally, the device’s production process should be rapid, scalable, and cost-effective, while also adhering to the regulations for a Class IIa medical device. In this phase, 3D-printing technology was chosen so that the design could easily be adapted to advancing insights. Notwithstanding, it was envisioned that the prototype was scalable and could be produced via injection modelling while maintaining its performance.

Several off-the-shelf valves were considered as well as the designs described by Levin and Sorg [21, 22]. However, none of which fully met the above design criteria, and we thus worked out a new concept to meet all design criteria and the intended use. This novel concept diverges from the existing valves commonly used in Flow Control Valves (FCV) and does not rely on the working principles of Pressure Gated Valves (PGV) [25, 26]. Therefore, the basic concept of FCV was revised and a prototyped device was designed.

Existing valves and prototyped devices were tested in the ISV bench setup (Fig. 2). All potential valves and devices were screened and tested for their potential to meet the design criteria. The delivered VT for each 10% valve opening increment was plotted and evaluated for its linearity as shown in Additional file 1: Figure S1. The ventilator setting was: an inspiratory pressure (IP) of 20 cmH_2_O, a PEEP of 5 cmH_2_O, a flow acceleration of 90 mbar/sec and FiO_2_ 0.21.

Various new device types and geometric variants were designed and fabricated using 3D printing technology. Of these, 2 types were eventually retained based on expert opinion and preliminary test results. These 2 types underwent further development and optimization through incremental changes aimed at enhancing device characteristics. The final focus was on achieving a linear effect in airflow manipulation while meeting all secondary design criteria.

**Fig. 3:**
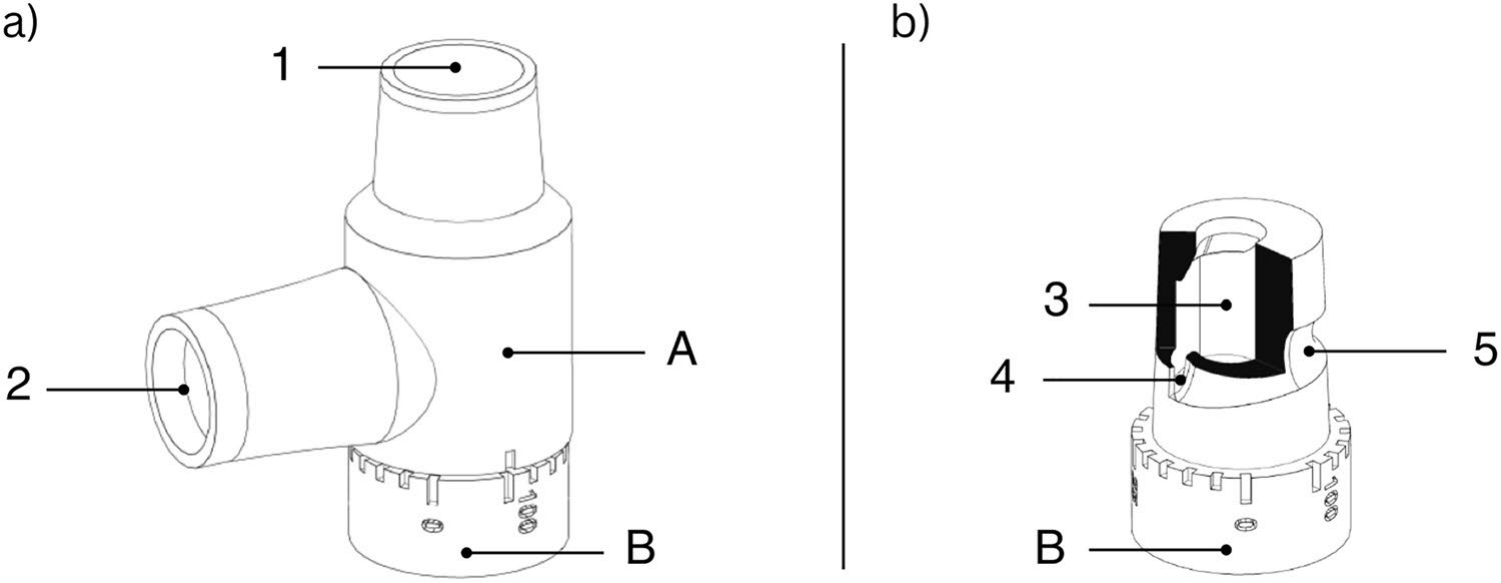
The flow modulator. a) The device comprises two main components: a body housing (A) equipped with an inlet (1) and an outlet (2), and a valve (B). The valve is cylindrical in shape, with taper, and rotates around its longitudinal axis within the housing (A). b) The airflow is captured (3) and tangentially directed through an aperture (4) towards a spirally arranged three-dimensional groove (5) located on the circumferential side wall of the valve body. A scale ranging from 0 to 100% (B) is marked on the device, indicating the transition from fully closed to fully open. Depending on the inspiratory pressure (IP) and lung characteristics, the position of the valve (B) relative to the housing (A) will deliver a predictable tidal volume (VT)

Ultimately, one specific design was selected based on its linear response, desired range of VTs, and ease of manufacturability (Fig. 3). Subsequently, the final prototype underwent performance testing to evaluate its suitability for the intended use, utilizing an in vitro bench setup.

### Bench-test of the prototyped device

The primary aim of the experimental bench study was to evaluate the performance of the prototyped device, where performance is defined as the accurate and independent delivery of VTs with limited incremental steps in the ISV circuit. This performance was assessed using two evidence-based simulated lung pathologies and various ventilator settings, as depicted in Fig. 2. Additionally, the pressure, flow, and volume waveforms were recorded to characterize specific mechanical ventilation properties.

### Different bench tests

1. Determine VTs and airway pressure (Paw) as function of percentage of the prototyped device opening by different set inspiratory pressures (IPs) for a test lung with normal and increased elastance.

The VT and Paw in both circuits were assessed at IPs of 20, 25, and 30 cmH_2_O in relation to the opening percentage of the prototyped device, with both artificial lungs set at the same elastance. This test was conducted at 10% intervals for each valve opening. The prototyped device in the contralateral circuit was set at a fixed 100% (fully open). The ventilator settings included a respiratory rate of 15/min, a flow acceleration of 90 mbar/s, and an inspiratory-to-expiratory (I:E) ratio of 1:2. The artificial lungs were configured as depicted in Fig. 2 for both normal and high elastance conditions.

(2) Determine the pressure, pressure drop, flow and volume profiles when using ISV in a test lung with normal and increased elastance.

The primary function of every ICU ventilator is to deliver a specific VT, establish a set PEEP, and FiO_2_ in either a pressure- or volume-controlled mode. The delivery of these ventilation parameters is associated with specific pressure, flow, and volume profiles within a predefined time interval. The core function of the prototyped device in ISV is to deliver predictable and accurate VTs in a pressure-controlled mode. The individualized VTs for each patient result from the interaction between pressure-controlled ventilation and the prototyped device, as influenced by airway resistance and lung compliance.

### Data analysis

Measurements of flow, pressure, and pressure drop were directly obtained, while additional ventilation parameters were computed using the Citrex H5 device. Each bench test was conducted over a series of 35 tidal cycles, yielding a low observed standard deviation that was considered clinically insignificant. Data visualization was carried out using the Python library Matplotlib. Subsequently, linear regression analyses were conducted using the Scipy library in Python. Two types of regression analyses were performed: one utilizing all available data and another excluding valve openings higher than 90%. The linear functions for these analyses were derived based on the intercept and slope obtained from the regression.

## Results

**Fig. 4:**
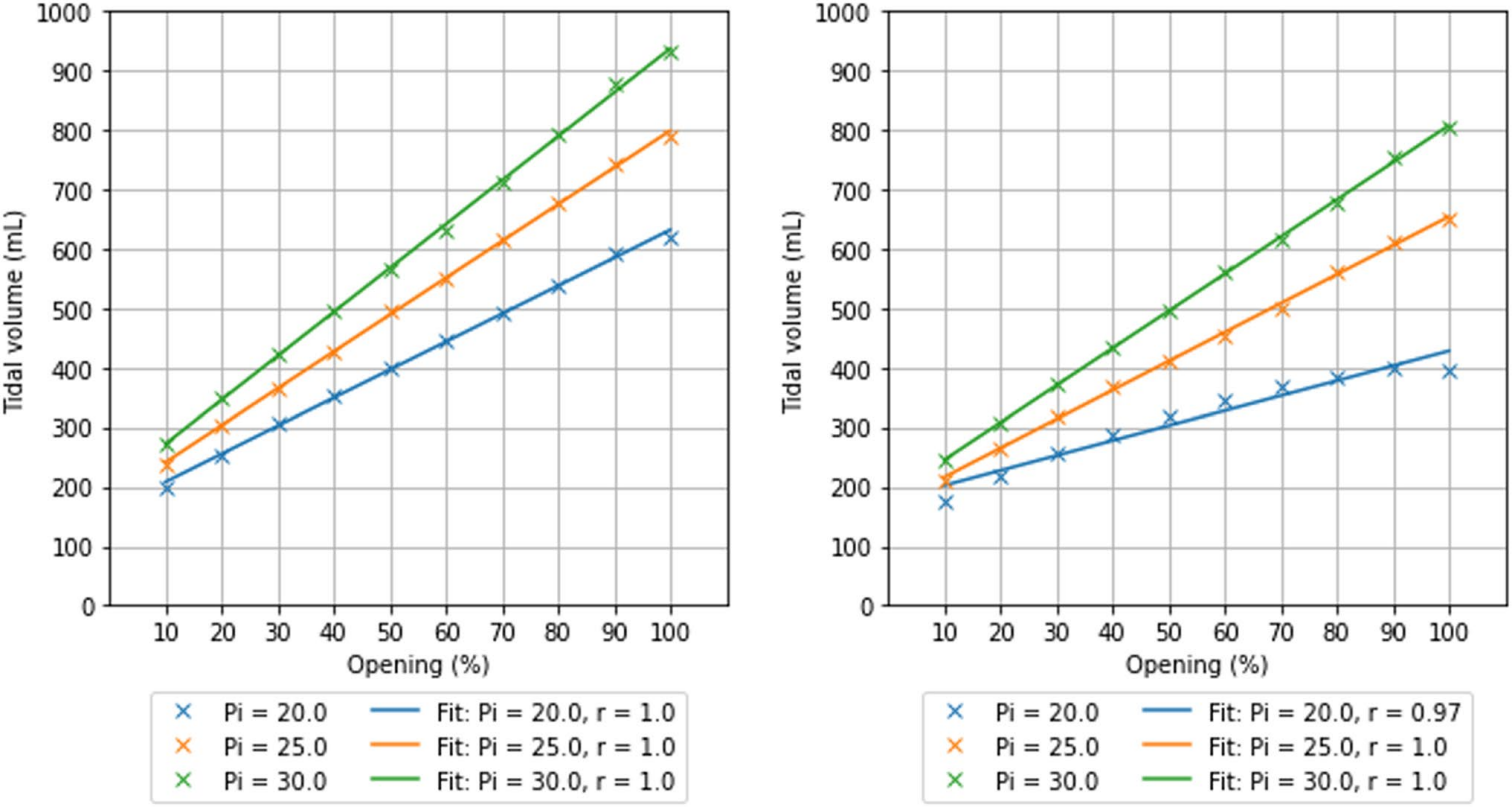
Tidal volume (VT) as function of the valve opening for different inspiratory pressures (IP). For both normal lung compliance (illustrated on the left) and low lung compliance (illustrated on the right), VT values were plotted against valve openings that ranged from 10–100%, at IPs of 20, 25, and 30 cmH_2_O. A linear fit was applied to the data points, and the Pearson’s correlation coefficient (r) is provided for each respective IP (*p* < 0.01)

### Performance

The prototyped device demonstrated a linear relationship within the clinically relevant VT range of 180 to 720 mL, as illustrated in Fig. 4. Linearity remained consistent even under conditions simulating high elastance test lungs. The Pearson’s correlation coefficient was 1.0 (*p* < 0.01) across all IPs within the 10–100% valve opening range for the low and high elastance test lung. Except for an IP of 20 cmH2O, the correlation coefficient was 0.97 (*p* < 0.01) in the high elastance test lung. When the valve opening range was confined between 10% and 90%, a correlation coefficient of 0.99 (*p* < 0.01) was obtained for the IP of 20 cmH_2_O, in the high elastance test lung.

A linear relationship was observed between the airway pressure and the valve opening across the three different levels of Inspiratory Pressure (IP), as illustrated in Additional File 1: Figure S2. This linearity was maintained in both normal and high compliance test lung settings, with the linearity being more pronounced in conditions of high elastance. The pressure within the circuit never surpassed the pressure settings configured on the ventilator (IP).

**Fig. 5:**
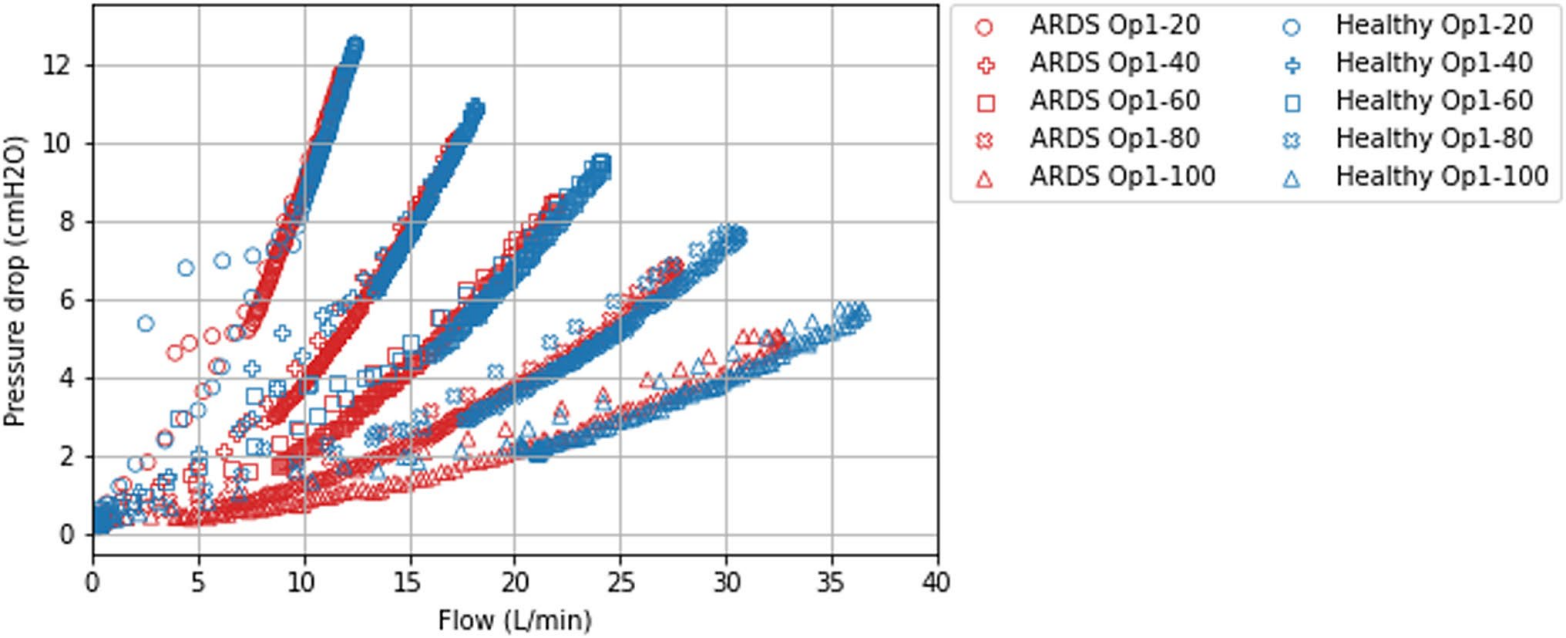
Pressure drop across the prototyped device as a function of flow during an ISV cycle. The pressure drop was plotted for various valve openings (20%, 40%, 60%, 80%, and 100%) under conditions of low and high lung elastance simulating both healthy (blue) and ARDS (red) lung pathologies. A quadratic relationship was observed between the pressure drop and flow. The pressure drop increased with rising flow, which was dependent on both valve opening and lung elastance. Conversely, the pressure drop decreased with increasing valve opening and increasing lung elastance

#### Pressure drop and flow across the prototyped device

The pressure drop across the prototyped device was inversely correlated with the valve opening (Fig. 5). An increase in pressure drop was associated with a decrease in airway pressure and reduced VT delivery (Fig. 4). Conversely, a decrease in pressure drop upon valve opening led to an increase in airway pressure, without surpassing the IP configured on the ventilator (Fig. S2).

The pressure drop across the prototyped device was also influenced by lung elastance, decreasing with increased elastance, as seen in ARDS conditions.

 Hence, the pressure drop across the prototyped device was affected by both the valve opening and lung compliance. At higher levels of lung elastance, the flow was observed to be lower. A similar reduction in flow was noted with smaller valve openings. A quadratic relationship was observed between the pressure drop across the prototyped device and the flow when operating in a pressure-controlled mode during ISV. This quadratic relationship persisted even when altering the valve opening with the prototype device. It was noted that the device induced variations in airway pressure as a function of both lung compliance and valve opening, even under pressure-controlled ISV ventilation conditions.

**Fig. 6:**
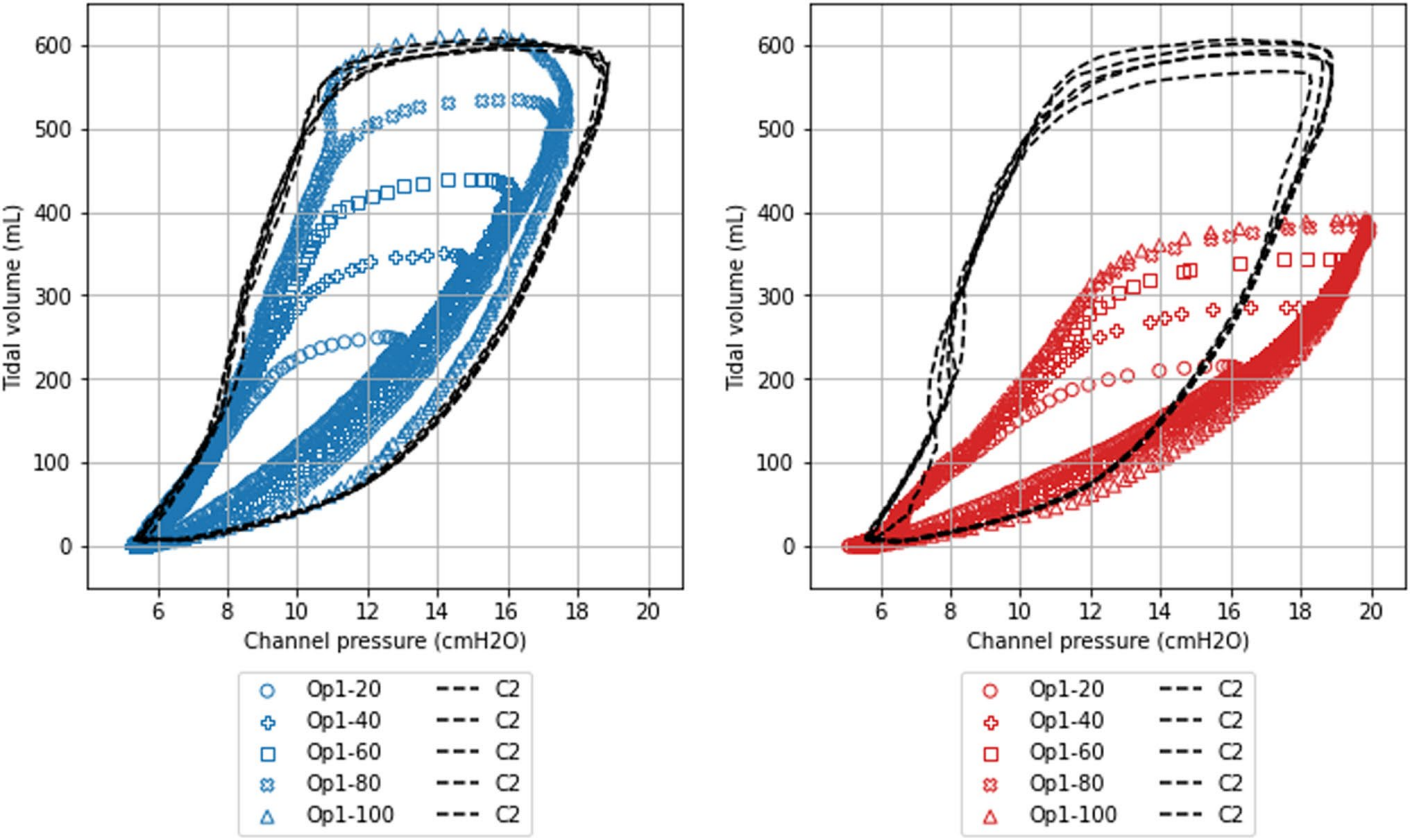
Pressure-Volume Loop of a Flow-Modulated Breath. The PV-loop was plotted for five valve openings (20%, 40%, 60%, 80%, and 100%) in Circuit 1 (solid line) and Circuit 2 (C2, dotted line) under conditions of low (blue) and high (red) lung elastance. The PV-loop shrinks with smaller valve openings, resulting in lower VTs. Under conditions of higher lung elastance, with consistent ventilator settings, the PV-loop also shrinks, leading to reduced VTs. Hence, when lung elastance increases, the delivered VT decreases, yet the pressure does not exceed the IP set on the ventilator

#### PV-loop

 In the circuit where the valve opening of the prototyped device was modulated within a range of 20–100%, both the pressure and the delivered VT were observed to decrease. (Fig. 6) In the contralateral circuit, where no modifications were made to the prototyped device, a stable delivered VT was maintained despite alterations in the neighboring circuit. A similar phenomenon was noted under high elastance conditions. In these conditions, the PV-loop, across varying valve openings but with consistent ventilator settings, produced a reduced VT.

**Fig. 7:**
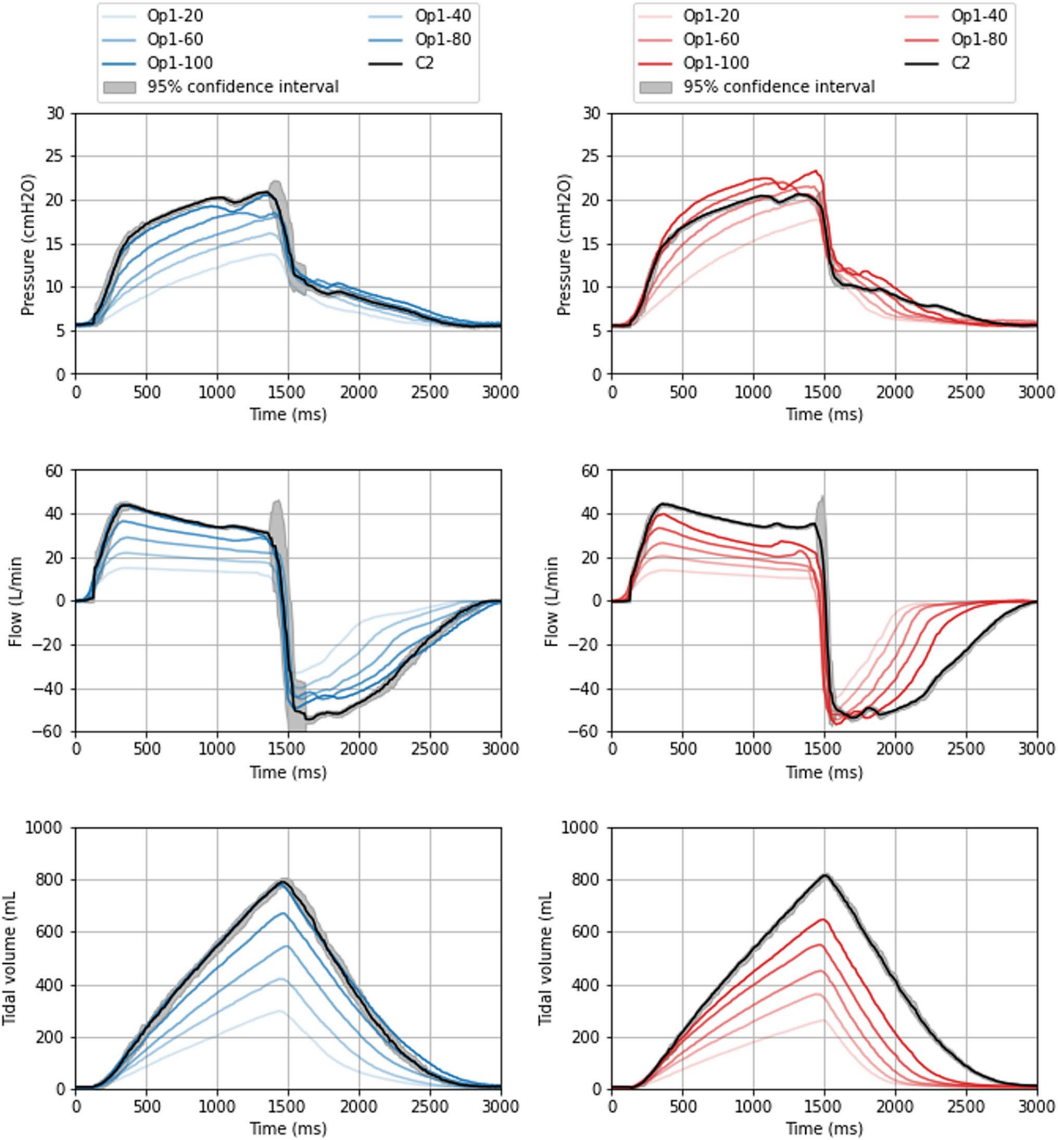
Time Evolution of Pressure, Flow, and VT for an ISV Cycle. The pressure, flow, and volume profiles are plotted for a normal (blue) and high (red) elastance test lung with an IP of 25 cmH_2_O across five valve openings (20, 40, 60, 80, and 100%). Circuit 2 is represented in black along with its 95% confidence interval, showing significant variation during the transition from inspiration to expiration. The ventilation profiles diverge from traditional modes of ventilation, with the volume profile resembling that of volume-controlled ventilation despite being pressure-controlled. The inspiratory phase of the flow profile features a plateau phase, a characteristic influenced by the prototyped device installed on the inspiratory circuit. The ISV profile is maintained across various valve openings as well as under different conditions of lung elastance

### ISV-breath

In our test circuit, an ICU ventilator operating in pressure-controlled mode was utilized. Despite this configuration, the observed ventilation profile in both the modulated and the unmodulated circuit diverged from conventional modes of ventilation, displaying features more commonly associated with volume-controlled ventilation. Figure 7 offers an in-depth illustration of the unique flow, pressure, and volume profile observed in the ISV circuit. The ventilation profiles for an IP of 20 and 30 cmH_2_O are shown in Additional file 1: Figure S3 and S4. The prototyped device was employed on the inspiratory circuit and resulted in a plateau phase during the inspiratory flow. These profile characteristics were maintained across various valve openings and under conditions of elevated elastance, such as in ARDS. Consistently, alterations to the valve opening in one circuit did not influence the other circuit. In Circuit 2, the 95% confidence interval was plotted, revealing variation primarily during the transition between inspiration and expiration.

## Discussion

We successfully developed a prototype of a valve that could produce a predictable and titratable VT in a shared ventilator setup, by modulating airflow. With the modification of the ventilation circuit, we observed a distinctive ventilation profile. The resulting ventilation profiles in both the flow-modulated and nonmodulated circuit did not match the typical pattern of a pressure-controlled ventilation mode, with different PV-loop and pressure, flow, and volume waveforms, indicating that this modified circuit has its own characteristics and limitations.

The potential to modify delivered TVs, and its novel ventilation pattern persisted in this bench testing in test lungs with normal and high elastance.

The prototyped device is a new concept of controlling VTs during mechanical ventilation, and was designed to offer temporary care during a ventilator surge capacity problem without the need of patient matching. The linear relationship between flow modulator opening and delivered VT for different inspiratory pressures and lung pathologies gives the potential to a high degree of predictability and accuracy. Prior to its application in clinical practice, this novel tool will need to be supplemented by a clinical protocol, which will guide the physician in delivering the desired ventilation parameters.

### Performance

The flow modulator had a linear relationship with the VTs delivered with the three different set inspiratory pressures and set test lung elastance. Furthermore, over the broad spectrum of flow modulation, small and predictable differences in resulting VTs could be achieved.

Flow modulation in one circuit did have an effect in the contralateral circuit. Although, when these results are framed in the light of a clinical situation, a high degree of independence is present without clinically relevant repercussions on the contralateral patient. A possible explanation is the integration of the flow modulator into the inspiratory limb of the circuit. Chatburn et al. described an alternative design to overcome this problem [30], by integrating valve into a splitter device. This does not meet the design criteria of intuitive ease of use by the operator and potentially introduce errors.

Previously, two major groups of devices to titrate VTs in ventilator sharing were described [26]. FCV reducing the cross-sectional area with different types of valves and PGV, which uses a spring load. Both operating principles involve setting and changing resistance, FCV by influencing the cross-section, PGV by influencing the spring load. Implementing this in a shared ventilator setup with pressure-controlled ventilation creates the possibility to individualize VTs. The flow resistance for PGV decreases with increasing flow and the pressure drop across the valve is independent from it [26]. The VT can therefore only be controlled by changing the pressure set by the valve or the ventilator and hence change the inspiratory pressure of the patient.

Flow across a channel with reduced cross-sectional area can on the other hand be characterized by the Darcy-Weisbach equation:

1 $$\Delta p = f\rho \frac{L}{D}\frac{{v^{2} }}{2},$$

where by ${\Delta }p$ is the pressure-loss, $f$ the Darcy-Weisbach friction coefficient, $\rho$ the mass density of air, L the length of the channel *D* the hydraulic diameter of the channel (i.e. the ratio of four times its cross-sectional area *A* to its perimeter) and *v* the average speed of the flow [32]. Depending on the flow regime, several empirical correlations have been proposed for the Darcy-Weisbach friction coefficient in Eq. 1. The turbine driven incompressible, unsteady oscillating flow reaching the flow modulator is of turbulent nature given the bellow tubing and numerous junctions, connectors, and filters on its pathway. Over the short length of the flow modulator and neglecting the effect of gravity, Bernoulli’s principle can be rewritten to yield a bounding value on the pressure-loss.

2 $$\Delta p = \frac{1}{2}\rho \frac{{Q_{{\max }}^{2} }}{{A^{2} }}~$$

Hence the existence of a quadratic and inherent relationship between the pressure-loss across a flow valve at constant opening and the flow for a steady flow, as opposed to an other theoretical hypothesis proposing a linear relationship [26]. Our bench data supports the quadratic relationship between flow and pressure drop when influencing the cross-sectional diameter (Fig. 5).

### Mechanical power

The mechanical power is an overarching variable which includes the other mechanical ventilation parameters: VT, plateau pressure, PEEP, and flow, which can lead to VILI. Our novel flow profiles had a pronounced plateau phase during inspiration. These profiles were associated with low mechanical power and energy delivery to the lung tissue during mechanical ventilation [33]. As a result, the flow modulator may enable further research into lung protective ventilation by flow modulation on the expiratory circuit or both to minimize mechanical power during mechanical ventilation [34]. 

### Integration of the prototyped device into ISV: validation and ISV-protocol

A shared ventilator setup is a clinical process and not just a mechanical act of splitting a ventilator with a modified circuit. The limited real-world experience with shared ventilation highlights the critical importance of a clinical protocol [35]. Furthermore, Raredon et al. stated that there are two major criteria to provide safe and reliable care when performing ventilator sharing: predictable control and patient independence [26]. 

The ISV technology can be further developed to be applied safely. For this, the development of a clinical shared ventilator protocol is indispensable in which the performance is documented, and the operator can correctly set up and handle the ICU ventilator and flow modulator. The flow modulator and clinical protocol could be a part of the arsenal for healthcare workers in the domain of pre-hospital care, emergency medicine, and intensive care, when they are confronted with a ventilator surge capacity problem.

The performance of the flow modulator and properties of the ISV waveform suggest the end of the shared ventilation paradigm. The idea of ventilating two patients in a pressure control mode with a modified circuit should switch towards providing ISV with the flow modulator using an ISV protocol. ISV is a new ventilation technique to provide individual respiratory support when a ventilator surge capacity problem occurs. Clinical implementation will depend on developing an ISV protocol and validating the performance of the flow modulator in ISV against the intended use of a ventilator surge capacity problem in bench and in vivo.

### Limitations

Ultimately, the shared ventilator setup is always inferior to a single ventilator per patient setting. We aimed to develop a device that could allow the clinician to temporarily mitigate some risks during a ventilator surge capacity setting. This study focused on developing a new concept of flow modulation, by generating modifiable and predictable VTs in a shared ventilator setup without patient matching. This is a necessary step to facilitate the further development of a safe and clinically usable shared ventilator setup.

Further scenarios need to be evaluated prior to a clinical implementation. First, we still need to fully evaluate the impact of additional PEEP modulation. During brief testing in our setup, the added PEEP valve did not affect the expiratory flow on the other ventilator circuit or did it impact inspiratory flow on any circuit. Second, performance of the prototype in other ventilator settings needs to be examined. It seems that the effect of the flow modulator can be bypassed by a long inspiratory time, where an equilibrium settles on the inlet and outlet of the prototyped device. When integrating and testing the prototyped device into the ISV system, the limits of inspiratory time should be explored and established. In this study, individualization of FiO_2_ was not examined with the flow modulator; however, based on our prior research, an additional sidestream of 100% oxygen in the shared ventilator circuit may facilitate this [16]. 

In the bench testing, only one ICU ventilator, Dräger Savina 300, was used. When validating the prototype, its performance should be tested on different ICU ventilators. It was already shown that even within ventilators there is a difference in performance, which will correlate with the ISV technology [36]. This highlights the importance of an ISV protocol that can encompass these differences.

The artificial lungs we used are limited in simulating lung pathologies with three different lung compliances and four different airway resistances. This limits us in simulating categorical lung pathologies. A more sophisticated lung simulator in which a spectrum of airway resistances and lung compliances could be set is needed to further evaluate the limitations of this technology.

One further limitation of a shared ventilator, in the setting of ventilator surge capacity, is the concurrent need for separate respiratory system monitoring. Patient safety is currently only guaranteed when individual VTs and delivered pressures are measured. However, with the performance of the flow modulator and the development of a clinical ventilation protocol, we hope to eventually reduce the need for advanced monitoring, when software models can accurately predict the individual patient’s VTs with minimal input from a monitor.

## Conclusions

Almost four years after the start of the COVID-19 pandemic, there is still an unmet medical need for a medically graded solution to tackle a ventilator surge capacity problem. We present the design of a flow modulator device to titrate VTs in a shared ventilator setup without patient matching. Its performance was tested in a bench setting, whereby the flow modulator can deliver independent, titratable and accurate VTs for different settings of airway resistance and lung compliance. The flow modulation results in a new characteristic ventilation profile.

The development of the flow modulator enables further development of shared ventilator setup technology and the development of a clinical protocol facilitating its clinical use during a ventilator surge capacity problem.

### Supplementary Information

Below is the link to the electronic supplementary material.

Supplementary material 1 (DOCX 376.3 kb)

## Data Availability

The datasets used and/or analyzed during the current study are available from the corresponding author on reasonable request.
