# Supplementary material for: Design of a flow modulation device to facilitate individualized ventilation in a shared ventilator setup
Source: J Clin Monit Comput. 2024 Apr 1;38(3):679–90. doi: 10.1007/s10877-024-01138-1 (PMC11164813; doi:10.1007/s10877-024-01138-1)
Supplement: Supplementary file 1 — Supplementary material 1 (DOCX 376.3 kb) [file 10877_2024_1138_MOESM1_ESM.docx]

**Supplemental legends**


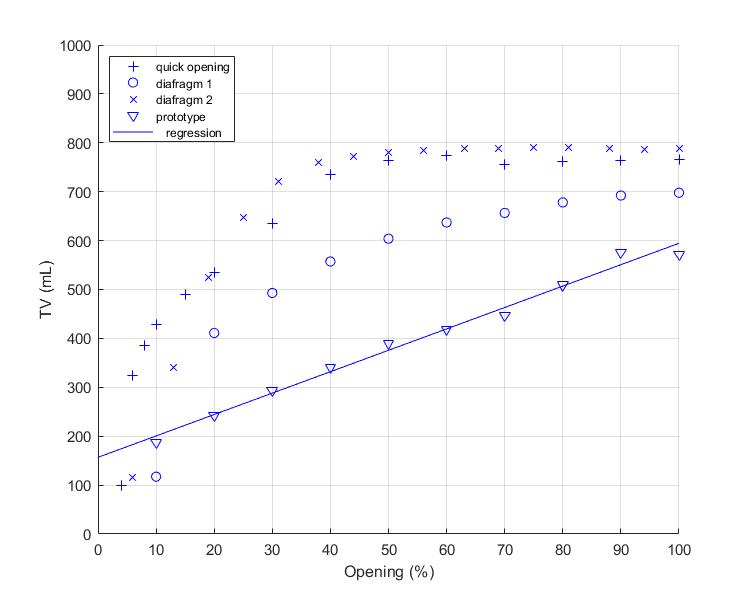


**Fig. S1: Tidal Volume (VT) in relation to valve opening across different valve types.** Diaphragm valves and quick-opening valves exhibit constant VT near full opening (100%), followed by a sharp decline when more than half-closed (<50%). These valves yield high VTs even at minimal openings, complicating control over the lower range. While some linearity is observed in their characteristics, the gradient varies significantly, particularly for the Diaphragm 1 type. This contrasts with the flow modulator (prototype), which demonstrates consistent linearity and uniform incremental steps in VT across its operational range.


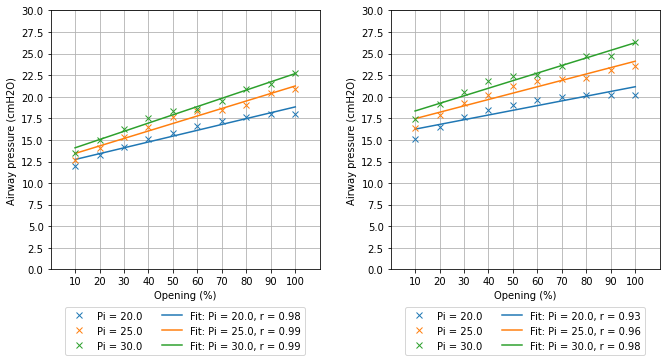


Fig. S2 **Airway pressure (Paw) as function of the valve opening for different inspiratory pressures (IP)**. For both healthy lung conditions (illustrated on the left) and Acute Respiratory Distress Syndrome (ARDS) pathology (illustrated on the right), Paw values were plotted against valve openings that ranged from 10% to 100%, at IPs of 20, 25, and 30 cmH_2_O. A linear fit was applied to the data points, and the Pearson’s correlation coefficient (r) (p < 0,01) is provided for each respective Paw.


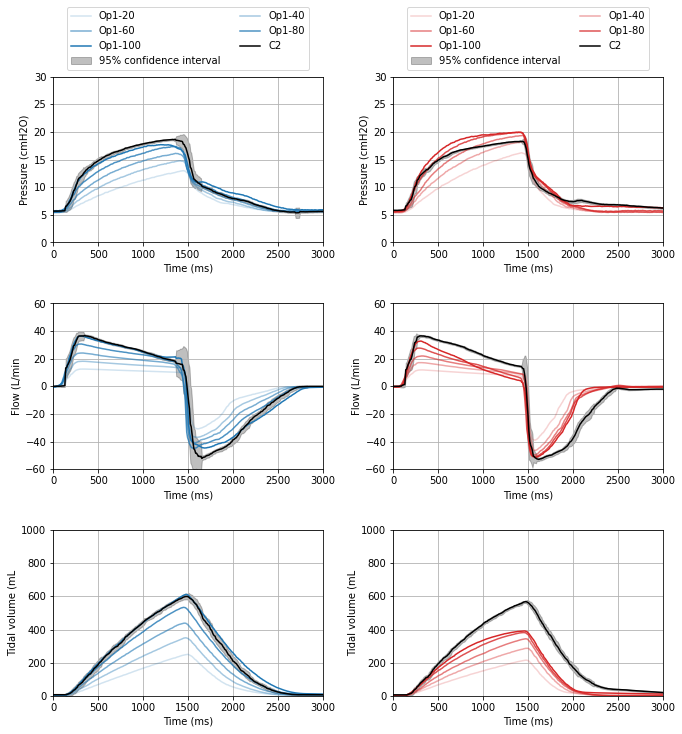


**Fig. S3 Time Evolution of Pressure, Flow, and VT for an ISV Cycle.** The pressure, flow, and volume profiles are plotted for a normal (blue) and high (red) elastance test lung with an IP of 20 cmH_2_O across five valve openings (20%, 40%, 60%, 80%, and 100%). Circuit 2 is represented in black along with its 95% confidence interval, showing significant variation during the transition from inspiration to expiration. The ventilation profiles diverge from traditional modes of ventilation, with the volume profile resembling that of volume-controlled ventilation despite being pressure-controlled. The inspiratory phase of the flow profile features a plateau phase, a characteristic influenced by the prototyped device installed on the inspiratory circuit. The ISV profile is maintained across various valve openings as well as under different conditions of lung elastance.


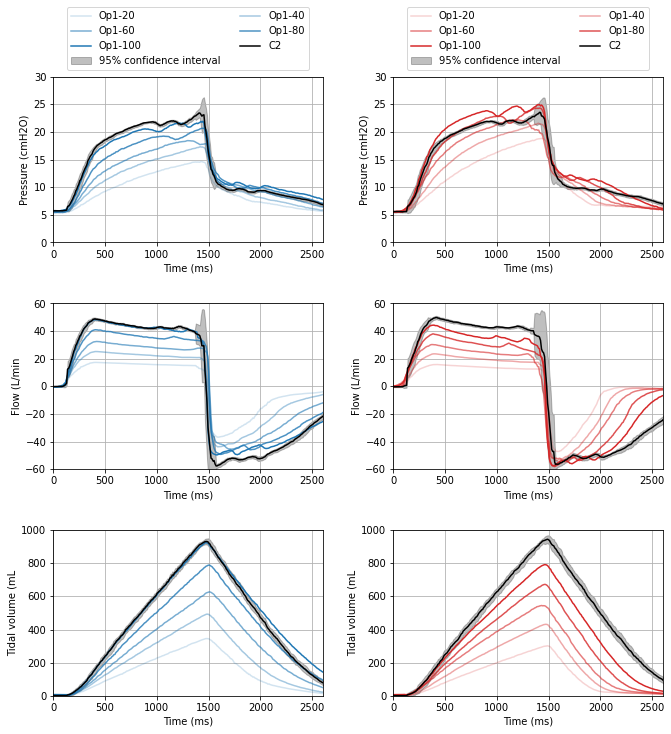


**Fig. S4 Time Evolution of Pressure, Flow, and VT for an ISV Cycle.** The pressure, flow, and volume profiles are plotted for a normal (blue) and high (red) elastance test lung with an IP of 30 cmH_2_O across five valve openings (20%, 40%, 60%, 80%, and 100%). Circuit 2 is represented in black along with its 95% confidence interval, showing significant variation during the transition from inspiration to expiration. The ventilation profiles diverge from traditional modes of ventilation, with the volume profile resembling that of volume-controlled ventilation despite being pressure-controlled. The inspiratory phase of the flow profile features a plateau phase, a characteristic influenced by the prototyped device installed on the inspiratory circuit. The ISV profile is maintained across various valve openings as well as under different conditions of lung elastance.
